# Processing–Microstructure–Property Relationships in a Cu-Rich FeCrMnNiAl High-Entropy Alloy Fabricated by Laser and Electron Beam Powder Bed Fusion

**DOI:** 10.3390/ma19061174

**Published:** 2026-03-17

**Authors:** David Maximilian Diebel, Thomas Wegener, Zhengfei Hu, Thomas Niendorf

**Affiliations:** 1 School of Material Science and Engineering, Tongji University, 1239 Siping Road, Shanghai 200092, China; 2 Institute of Materials Engineering—Metallic Materials, University of Kassel, Mönchebergstraße 3, 34125 Kassel, Germany; thomas.wegener@tu-darmstadt.de (T.W.); niendorf@uni-kassel.de (T.N.)

**Keywords:** high-entropy alloy, medium-entropy alloy, additive manufacturing, FCC, BCC, dual phase

## Abstract

A Cu-containing FeCrMnNiAl multi-principal element alloy was processed by laser-based and electron beam-based powder bed fusion (PBF-LB/M and PBF-EB/M) to investigate processing–microstructure–property relationships. In focus were alloy variants with a relatively high Cu content. Two PBF-LB/M scan strategies, employing a Gaussian beam with and without a re-scan with a laser featuring a flat-top profile, were compared to PBF-EB/M processing, followed by heat-treatments between 300 °C and 1000 °C. The phase constitution, elemental partitioning and grain boundary characteristics were analyzed by X-ray diffraction, electron backscatter diffraction and energy-dispersive X-ray spectroscopy. Mechanical behavior was assessed by hardness and tensile testing. Both manufacturing routes promoted the evolution of stable multi-phase microstructures composed of face-centered-cubic (FCC)- and body-centered-cubic (BCC)-type phases across all heat-treatment conditions. PBF-LB/M processing resulted in finer, dendritic microstructures and suppressed formation of a Cu-rich FCC phase due to higher cooling rates, whereas PBF-EB/M promoted the evolution of Cu-rich FCC segregates and equiaxed grain morphologies. Heat-treatment above 700 °C led to recrystallization, accompanied by an increase of the FCC phase fraction, grain coarsening, and recovery. At lower heat-treatment temperatures, the changes in microstructure are different. Here, it is assumed that small, non-clustered Cu-rich precipitates formed at the grain and sub-grain boundaries, although this assumption is only based on the assessment of the mechanical properties. The size of these precipitates is below the resolution limit of the techniques applied for analysis in the present work. Additional structures seen within the Cu-rich areas of PBF-EB/M-manufactured samples treated at lower temperatures also seem to have an influence on the hardness and yield strength. All of the conditions investigated exhibited pronounced brittleness, limiting reliable tensile property evaluation and indicating the need for further optimization of processing strategies and microstructural control for high-Cu-fraction-containing multi-principal element alloys.

## 1. Introduction

Based on one of the first reported high-entropy alloy (HEA) compositions by Cantor [1], researchers have attempted to replace costly elements such as cobalt (Co) with more cost-effective alternatives [2]. One element considered for substitution is copper (Cu), aiming to enable more economically viable compositions, or the use of recycled feedstock [3,4]. Former studies reported that adding Cu can promote the formation of a face-centered cubic (FCC) phase and refine the grain structure [5]. However, higher Cu additions in Cantor-based compositions can also lead to segregation and the formation of a Cu-rich FCC phase, driven by the mixing enthalpy between Cu and other elements [3,4,5]. Such Cu-rich segregation and increased Cu content have been reported to affect both mechanical and corrosion properties. Depending on the specific composition, reduced corrosion resistance and decreased mechanical performance have been observed [4,5]. Consequently, different compositional approaches have been exploited to mitigate Cu segregation, for example by reducing the Cu fraction [3] or by adding elements like manganese (Mn) [2]. Mn additions have also been associated with the formation of body-centered-cubic (BCC)-type phases and increased hardness. Moreover, studies on other HEAs indicate that processing routes with high cooling rates, such as laser-based powder bed fusion of metals (PBF-LB/M), can suppress equilibrium phase formation [6]. In current applications, different laser profiles are utilized to optimize the process. Among them, the Gaussian and flat-top (also named tophat) laser profiles have demonstrated significant differences in intensity distribution across their beam cross-section [7]. While the Gaussian laser profile shows a centered peak intensity that is higher than the average, the flat-top laser profile demonstrates a more uniform, averaged intensity without central hot spots [7]. The arising thermal stresses are therefore more pronounced for the Gaussian laser profile, characterized by a higher thermal gradient, while the flat-top profile and its more uniform heat across the scanned area promotes less thermal stress due to its lower thermal gradient [7].

To the best of the authors’ knowledge, only a few studies have investigated Cantor-derived HEAs with elemental combinations in the FeCrMnNiAlCu system [2,3,4,8,9]. Small aluminum (Al) additions are expected to promote BCC-type phase formation and thereby increase strength [3,5], whereas a higher Cu content is frequently associated with the segregation of a Cu-rich FCC phase and potentially reduced mechanical and corrosion properties, as elaborated above [4,8]. As also mentioned before, Mn additions have been suggested to mitigate Cu segregation and to further promote BCC-type phase formation [2]. However, the influence of the manufacturing route has not been systematically addressed for these alloys. In particular, tensile data for high-Cu variants processed by PBF-LB/M remain limited in open literature, as well as the impact of using different laser profiles like Gaussian or flat-top in PBF-LB/M.

In order to close this prevalent research gap, the present study investigates a novel FeCrMnNiAlCu composition with a high Cu content. By comparing different manufacturing routes—PBF-LB/M and electron beam-based powder bed fusion (PBF-EB/M)—along with post-processing heat-treatments, their respective influences on the microstructural evolution and mechanical properties are assessed. For PBF-LB/M, two process variations are examined: (i) a condition only processed by a low-power Gaussian-profile laser and (ii) the same strategy combined with an additional re-scan using a high-power, flat-top laser. By comparing these processing routes before and after subsequent heat-treatment, the potential effects on Cu segregation can be evaluated. Assessment of PBF-EB/M samples with the subsequent heat-treatments further broadens the perspective of process–microstructure relationships. Overall, the results provide a first step toward mitigating the pronounced brittleness of the material and support the future optimization of multifunctional, HEA compositions containing high fractions of Cu.

## 2. Material and Methods

To identify potential heat-treatment conditions, CALPHAD simulations were performed using Thermo-Calc 2023b in combination with the SSOL7: SGTE Alloy Solutions v7.0 database. For sample fabrication by PBF-LB/M and PBF-EB/M, pre-alloyed powder with a particle size distribution of 53–150 μm was employed. The powder composition was verified by chemical analysis and is summarized in Table 1.

PBF-LB/M samples were manufactured on an SLM 280^HL^ system (SLM Solutions, Lübeck, Germany) equipped with a 400 W Gaussian-profile laser and a 1 kW flat-top laser. Two processing strategies were investigated: (i) single exposure using the Gaussian beam (P17) and (ii) Gaussian exposure followed by an additional flat-top-profiled laser re-scan. The applied process parameters are listed in Table 2. A hatch distance of 70 μm, a layer thickness of 80 μm, and a constant substrate preheating temperature of 700 °C were used for all builds.

PBF-EB/M samples were produced on an Arcam A2X system (Arcam AB, Mölndal, Sweden). The corresponding process parameters are provided in Table 3. A hatch distance of 70 μm and a layer thickness of 80 μm were applied. Prior to melting, the powder bed was sintered at 950–1050 °C for 45 min. Preheating parameters included 5 mA beam current, a 12,000 mm/s beam speed and 30 repetitions. All of the parameters for both manufacturing processes result from an internal parameter study aimed at achieving sufficient densification of at least 99.6%.

**Figure 1:**
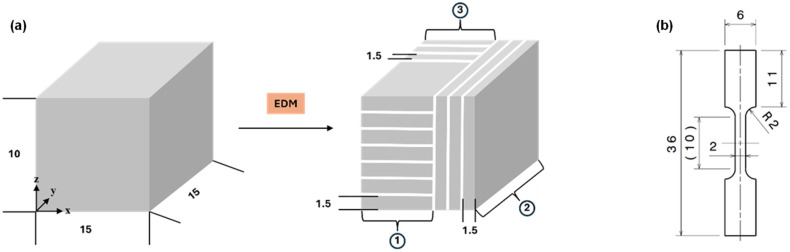
Pattern and final shape of (**a**) cubic samples processed (with *z*-axis parallel to building direction) and (**b**) samples machined for tensile testing by electrical discharge machining. The loading direction of the samples corresponds to the *y*-axis in (**a**). All dimensions are given in mm.

The test sets comprised cubic samples with dimensions of 15 × 15 × 10 mm^3^ and additionally manufactured, tensile samples extracted by electrical discharge machining (EDM), as illustrated in Figure 1. Prior to mechanical testing, the cubic samples were sectioned into 1.5 mm thick slices and heat-treated in an ambient atmosphere at 300 °C, 500 °C, 700 °C, 800 °C, 900 °C and 1000 °C for 8 h, followed by water quenching. For each build orientation (x–y plane and z-direction), three slices were selected to assess depth-dependent hardness evolution after heat-treatment (see ① and ②, ③ in Figure 1a). The samples have been ground by P2500 or P4000 and subsequently characterized using a Keyence VHX-600 (KEYENCE, Neu-Isenburg, Germany) digital optical microscope. The gathered images of all slices have then been processed by Image J 1.54 g, formatted in 8-bit, and analyzed by the function threshold to assess their density.

Vickers hardness measurements were performed using a LECO V-100-C1 tester (Leco Corporation, St. Joseph, MI, USA) with a load of 9.8 N and a dwell time of 15 s. Phase analysis was conducted by X-ray diffraction using an Empyrean X-ray diffractometer (Panalytical GmbH, Kassel, Germany) equipped with a Cu-Kα source operating at 40 kV. The measurements were conducted for a 2θ scanning range of 40–150°, with a step size and acquisition time of 0.05° and 8 s, respectively.

Four tensile samples were heat-treated under identical conditions to the cubic slices and tested at room temperature using a screw-driven MTS Criterion load frame (MTS Systems Corporation, Eden Prairie, MN, USA). A strain rate of 0.18 mm/min was applied. Strain was recorded using an MTS miniature extensometer (MTS Systems Corporation, Eden Prairie, MN, USA) with a gauge length of 5 mm and a strain limit of 30%. To minimize surface effects, tensile samples were machined by removing 1 mm from each surface via EDM, followed by a final low-speed cutting step to obtain a 1.5 mm thick testing geometry without additional grinding or polishing. Consequently, the evaluated mechanical properties reflect conditions closer to economical industrial applications, where complete surface finishing is not always feasible.

For in-depth microstructural characterization, a Zeiss Ultra Gemini field-emission scanning electron microscope (SEM) (Carl Zeiss AG, Oberkochen, Germany) equipped with electron backscatter diffraction (EBSD) and energy-dispersive X-ray spectroscopy (EDS) detectors was employed. The EDS measurements were carried out at an acceleration voltage of 20 kV using magnifications of 3000× and 12.5k×. The EBSD analyses were performed at 20 kV with magnifications between 1000× and 2000× as well as 12.5k×. Prior to microscopy, all samples were mechanically ground to 5 μm using SiC paper and subsequently vibro-polished for 24 h with a colloidal silica suspension with a 0.06 μm particle size. The EBSD images presented in the present work always include the image quality (IQ) parameter obtained during EBSD acquisition. Phase discrimination and further quantitative evaluations were carried out using the indexed phase maps. Therefore, grain boundary rotation angles below 2° have been set to be disregarded and were not taken into account for the evaluation. No further data smoothing was carried out. The kernel average misorientation (KAM) maps were generated using OIM 7.3 software. The misorientation was thereby calculated based on the first nearest neighbor, with a maximum misorientation threshold of 5°. The analysis included all points within the kernel, and 0-point kernels were assigned the maximum misorientation value to avoid data gaps.

## 3. Results and Discussion

**Figure 2:**
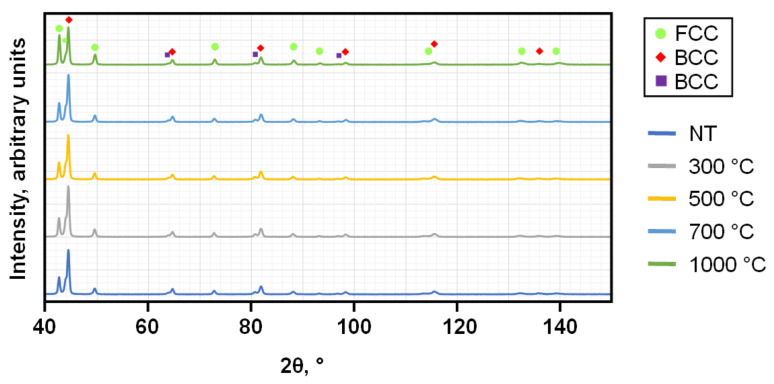
XRD results of non- and post-process heat-treated PBF-LB/M P17 samples.

Before examining the differences between the PBF-LB/M- and PBF-EB/M-manufactured samples, the results obtained from the two different PBF-LB/M procedures are discussed separately and then considered from a shared perspective, adding those of the PBF-EB/M condition. Generally, it is assumed that the complex interplay of different elemental mechanisms not only depends on the additive manufacturing (AM) process used, but also the subsequent heat-treatment. To support the EBSD investigations, EDS was used to examine the elemental distribution and possible segregations, while initial XRD measurements allow to gain an initial impression of the prevailing phases. Supported by knowledge gained from existing literature, the selection of the parameters for the EBSD phase analysis was conducted. Based on additional Scheil simulations with solute trapping, the microstructure resulting from the PBF-LB/M process is predicted to be a single FCC phase without intermetallic precipitation. The library used for simulation has not been adapted for HEAs, so experimental analysis is required to assess the quality of the predictions. Here, XRD investigation revealed that the microstructure of the PBF-LB/M-manufactured samples in both conditions is composed of FCC- and BCC-type crystal structures (see Figure 2). Subsequent heat-treatment has an influence on the ratio of the intensity peaks, but not on the corresponding peak positions, ultimately indicating a stable multi-phase structure without emerging or dissolving phases.

**Figure 3:**
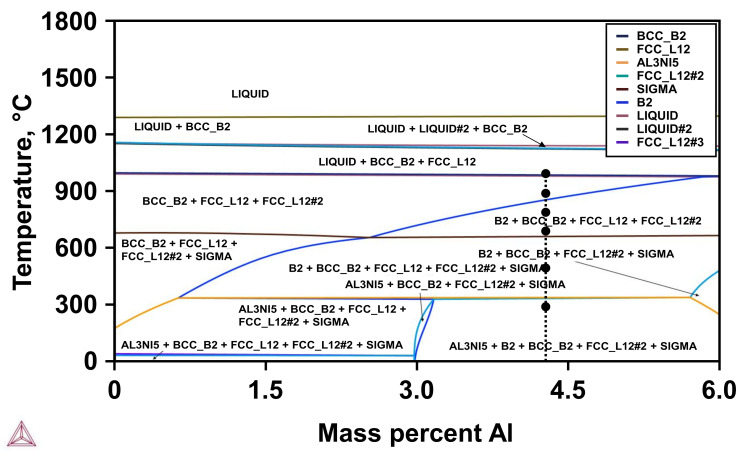
Calculated phase diagram of FeCrMnNiAlCu. See text for details.

By comparing these results to the calculated phase diagram (see Figure 3), additional differences between the theoretical predictions and experimental results can be derived.

**Figure 4:**
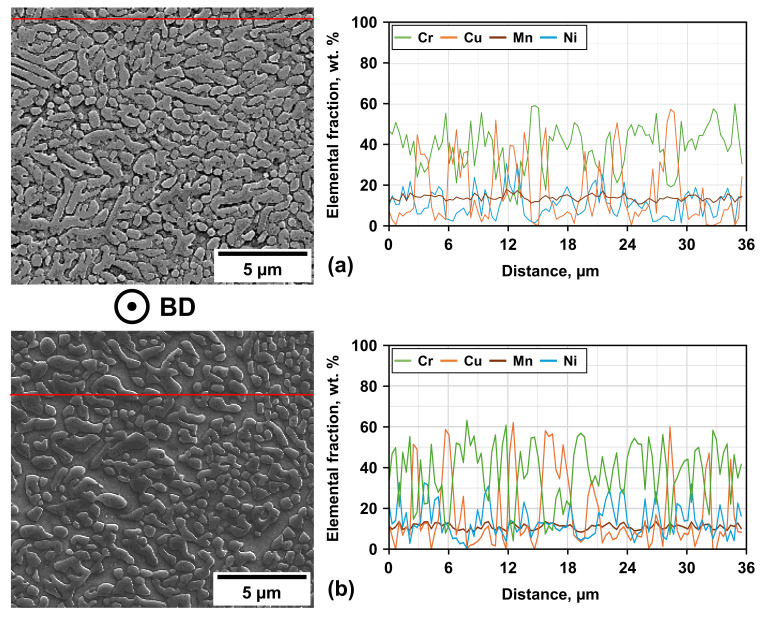
SE pictures and results of EDS line scans for PBF-LB/M-manufactured (**a**) NT samples and (**b**) samples post-AM heat-treated at 900 °C. Level of used line is marked in red on SE image (differing scale).

Partial liquefaction for samples treated at 1000 °C could not be confirmed, but observations reveal a multitude of FCC and BCC phases. While the exact phase constitution cannot be evaluated by the applied XRD analysis alone, the presence of the Sigma phase could not be shown for the sample heat-treatment at 500 °C. EDS line scans have been conducted to investigate the elemental distribution and inherent segregation patterns upon different heat-treatments (see Figure 4). Additional evaluation of the secondary electron (SE) images points towards the specific grain morphology of the samples. At this point, it should be noted that non-heat-treated samples are referred to as NT throughout the remainder of the manuscript.

The NT samples manufactured by PBF-LB/M demonstrate an elongated, dendritic-like grain morphology with ultra-fine-grained substructures. The EDS line scans pinpoint three distinct areas of enrichment of certain elements, while Mn seems to be distributed almost homogeneously. The dendritic regions are enriched by Fe-Cr and the interdendritic regions contain more Cu. In reference to former research detailed in [3], it is thought that the Fe-Cr-enriched dendritic region should be BCC (A2). Furthermore, Ni-Al-rich regions composed of the BCC (B2) phase are expected; however, they can hardly be seen here. Regarding the Cu-enriched FCC phase within the interdendritic regions, [2] mentions that the addition of Mn into the alloy composition obstructs the segregation of Cu. Therefore, [3] reduced the Cu content. The Mn fraction in both former studies is higher than in the tested composition of the present work. This could be a reason for the EDS results obtained being characterized by combined peaks of Mn and Cu as well as the possible minor 2θ-angle deviation of the pure Cu peak compared to the evaluated XRD results of Figure 2. Another result of the homogeneous Mn distribution could be the peak appearing at a 2θ-angle between 43.975° and 44.025°. According to the analysis, this angle corresponds to an Al-Mn-Ni-rich FCC-type phase. The presence of this phase is reasonable according to [2], as EDS line scans of the interdendritic regions show higher contents of Al, Mn and Ni. Finally, the separated peaks seen at 2θ-angles around 64°, 80° and 96° have to be discussed. The authors in [5] suggest that such peak splitting may stem from lattice distortion, which they attribute to the coexistence of BCC- and B2-type phases caused by altered Cu content in similar HEAs.

**Figure 5:**
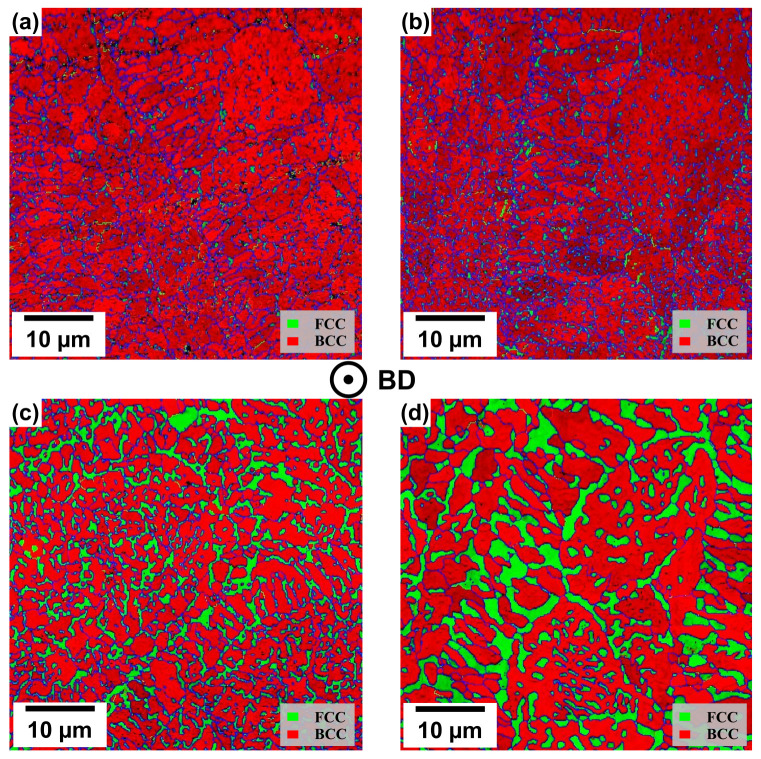
Phase distribution map of PBF-LB/M-manufactured FeCrMnNiAlCu. (**a**) P17 NT, (**b**) re-scan NT, (**c**) re-scan 700 °C post-treated and, (**d**) re-scan 900 °C post-treated samples. The blue lines highlight boundaries based on evaluated rotation angles ≥ 15°.

The phase distribution maps obtained by the EBSD investigations (see Figure 5) reveal that in the case of the NT samples in both PBF-LB/M process conditions, the Fe-Cr-rich BCC (A2) phase is dominant, while the other phases show only minor fractions. This ratio changes during heat-treatment at different temperatures, leading to an increase of the Cu-rich FCC phase accompanied by a similar decrease of the BCC (A2) phase (see Table 4). This can be explained by the higher cooling rate of the PBF-LB/M process leading to suppressed FCC-phase formation [6]. Additionally, the detected phases show only minor fractions and negligible changes upon post-treatments, so they are not shown here. By directly comparing the results of the NT samples to those of samples treated at 700 °C and 900 °C (see Figure 5c,d), it can be derived that the FCC phase tends to emerge at the grain boundaries of the dominating BCC (A2) phase [6].

**Figure 6:**
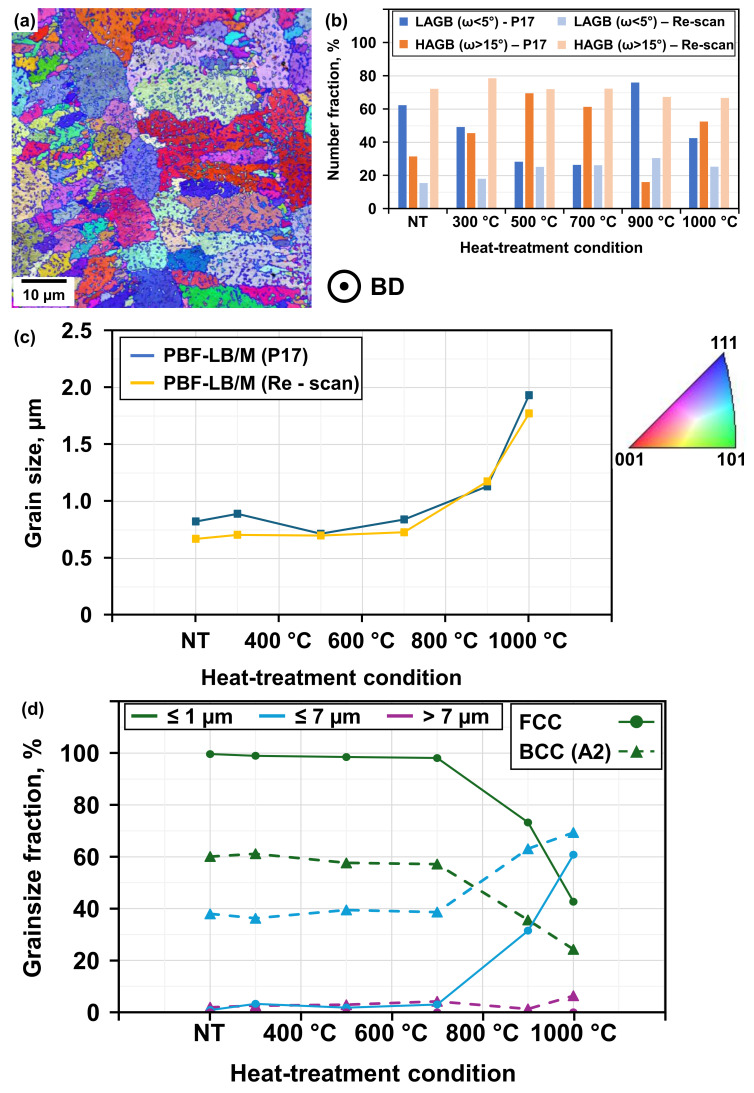
(**a**) Crystal orientation maps of PBF-LB/M-manufactured samples with a re-scanning procedure and heat-treatment at 700 °C, (**b**) angle of misorientation in the Cu-rich FCC phase (process-dependent), (**c**) average grain size, (**d**) phase-dependent grain size distribution following the re-scanning procedure.

Of note is the significant increase of the Cu-rich FCC phase between 700 °C and 900 °C, which is accompanied by signs of recrystallization, these signs being deduced from the appearance of the microstructure depicted in the crystal orientation map (see Figure 6a), i.e., related changes in grain size and morphology. The former grain morphology of long, columnar grains with nano-sized sub-grain structures dissolves, and both process conditions exhibit a similar grain size evolution (see Figure 6c). Interestingly, the BCC (A2) phase reveals a more balanced mixture of nano-sized (≤1 µm) and medium-sized grains (≤7 µm), while the FCC phase is characterized by a majority of approximately 97% nano-sized grains (see Figure 6d) [5]. Heat-treatment above 700 °C reveals pronounced grain coarsening for both phases, marking the onset of recrystallization, while recovery effects have already been observed at lower temperatures. The calculated misorientation between the FCC and BCC (A2) phases points towards the existence of a peak angle below 7°; the main peak angle for all variations and heat-treatment conditions is 43.04° [10]. In addition, a grain orientation spread (GOS) majority below 1° indicates only minor strain heterogeneities [11].

Assessment of the Pole Figures (PF) and Inverse Pole Figures (IPF) led to the conclusion that all of the tested samples do not show a distinct orientation relationship (OR), which has been mentioned in [10,11,12,13,14]. Furthermore, they do not show any texture or favored orientation. The tested samples show a majority of high-angle grain boundaries (HAGBs) throughout all heat-treatment conditions [15], with a minority demonstrating increased fractions of low-angle grain boundaries (LAGBs) within the examined FCC phase of the P17 process variation (see Figure 6b). Nevertheless, no obvious signs of twinning were observed in any tested sample [16]. This fact eliminates one of the possible strengthening mechanisms, discussed at a later point of this work. Based on [16], which reports that LAGBs may result from thermal stress due to rapid cooling within the PBF-LB/M process, it can be assumed that cooling rates affected by the presented process variations (different laser profiles) can ultimately influence the boundary character distribution. The results obtained indeed demonstrate the influence of different laser profiles. The presented evolution of LAGBs within the probed samples of the re-scanning variation (see Figure 6b) point towards a lower cooling rate, while the observations of the P17 variation are thought to be the result of a higher cooling rate (leading to increased thermal stress and thereby higher fractions of LAGBs). This might provide the rationale for another observation, i.e., the Cu-enriched FCC phase exhibits higher fractions of LAGBs than the Fe-Cr-enriched BCC phase (see Table 5).

The elemental distribution indeed indicates that the thermal conductivity within the FCC phase is higher than within the BCC (A2) phase, leading to higher thermal stress during PBF-LB/M manufacturing and heat-treatment. This hypothesis is supported by the results evaluated. Nevertheless, other expectation can be drawn from the existing literature [17,18,19]. These clearly point at the necessity of further experimental verification; however, this is out of the scope of the present work. As mentioned before, recrystallization has been observed in the heat-treated samples at around 700 °C, progressively reducing the dislocation substructures inherited from the PBF-LB/M process. While recovery can lead to rearranged dislocations forming sub-grain structures, i.e., LAGBs, recrystallization most likely results in HAGBs [18]. Nevertheless, depending on the stored energy, LAGBs can migrate into HAGBs even during recovery [18]. The results for samples of the P17 process variation show a decreasing fraction of LAGBs within the FCC phase for heat-treatments below 900 °C (see Figure 6b). The results concerning the microstructural evolution point towards clear differences between the two variations of the PBF-LB/M process. The fraction of the FCC phase within the NT samples of the re-scan variation is significantly higher than that of the P17 variation (see Table 4). Both conditions were manufactured with a constant substrate preheating of 700 °C, therefore the powder and manufactured samples have experienced the same intrinsic heat-treatment. Additionally, every layer was scanned twice in the re-scan condition and thus the process time is longer than for the P17 condition, enhancing the influence of the in-process heat-treatment. As both manufacturing variations used identical parameters for the Gaussian laser profile, the second scanning with the flat-top laser profile and the longer process time have most likely resulted in a less pronounced suppression of FCC phase formation as well as a lower stored energy and dislocation density.

Before comparing the mechanical properties of these two PBF-LB/M variations, the microstructural comparison is extended to include the PBF-EB/M process, thereby focusing on the discussion of the differences between the PBF-LB/M-manufactured samples of condition P17 and those of the PBF-EB/M process.

**Figure 7:**
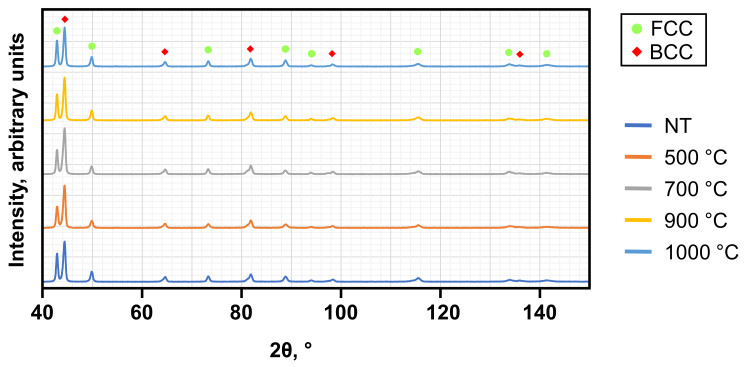
XRD results of non- and post-process heat-treated samples manufactured by PBF-EB/M.

A distinct difference between the processes is that the previously described peak splitting cannot be verified by XRD examinations of PBF-EB/M-manufactured samples (see Figure 7), indicating that the lattice distortion is less pronounced. This can be explained by the lower cooling rate within the PBF-EB/M manufacturing process [20].

**Figure 8:**
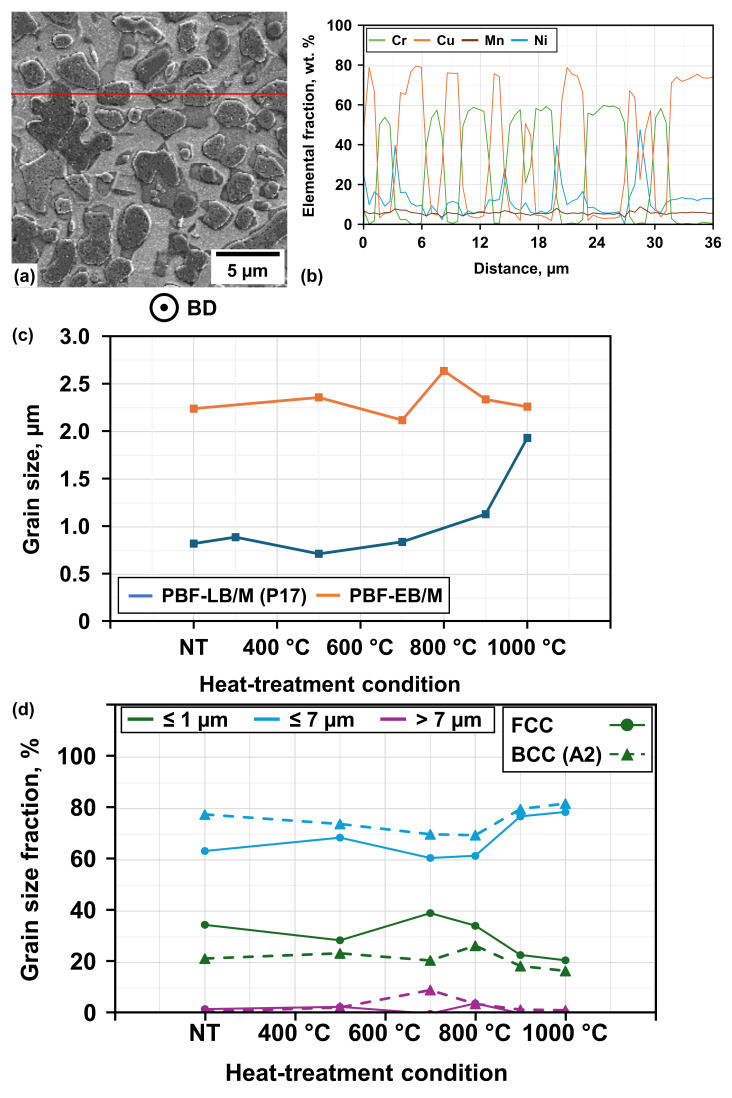
SE pictures and results of EDS line scans for (**a**) PBF-EB/M-manufactured samples with (**b**) elemental distribution in wt. %, (**c**) average grain size, and (**d**) PBF-EB/M phase-dependent grain size distribution. Level of used line is marked in red on SE image (differing scale).

Another result of the XRD investigation is that the observed 2θ positions are stable for the PBF-EB/M samples across all heat-treatment temperatures as well. This HEA composition should therefore not show emerging new phases within the tested AM processes with and without subsequent heat-treatments. Other similarities to the PBF-LB/M process can be noted when comparing the XRD results with the phase diagram of Figure 3. Heat-treatment at 1000 °C does not lead to a verification of partial liquification, but instead shows evolution of an FCC + BCC dual-phase microstructure. Heat-treatment at 500 °C did not show an emerging Sigma phase. Additional EDS line scans have been applied to examine the element distribution and potential segregations. SE images of the PBF-LB/M- (see Figure 4) and PBF-EB/M- (see Figure 8a) manufactured samples reveal a significantly different grain morphology, with the latter showing more equiaxed grains of increased size (see Figure 8c).

Based on the results of the EDS line scans (see Figure 8b), the previously mentioned three characteristic regions of the PBF-LB/M process can be identified even more clearly. The Cu-enriched phase is formed in a mesh-like morphology around island-like Fe-Cr-enriched grains, while the Ni-Al-enriched areas (detected here by localized enrichment of Ni) are located at their interfaces, occasionally reaching into the Cu-enriched regions. The majority of the probed sample areas exhibit a homogeneous distribution of Mn. Based on these observations and the former discussion, it can be assumed that the Fe-Cr-enriched regions correspond to a BCC (A2) phase, the Ni-Al-enriched regions to a BCC (B2) phase, and the Cu-enriched regions to a FCC-type phase. Potential shifts of the measured peaks from the database used can be explained by the influence of homogenously distributed Mn and the resulting lattice distortion [21].

**Figure 9:**
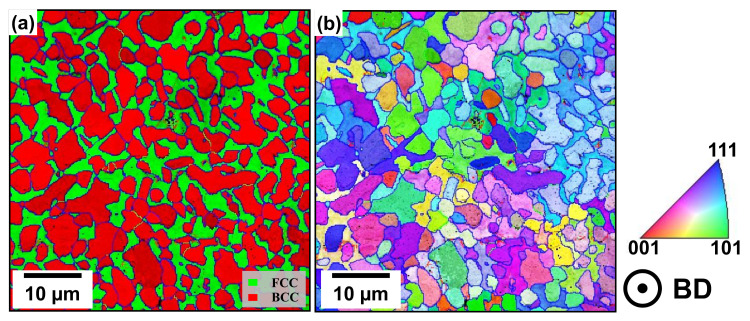
PBF-EB/M-manufactured FeCrMnNiAlCu investigated by EBSD. (**a**) Phase distribution map with blue lines highlighting boundaries based on evaluated rotation angles ≥ 15°. (**b**) Crystal orientation map for NT samples.

As already concluded for the PBF-LB/M-manufactured samples, EBSD investigations of the PBF-EB/M-manufactured samples show similar results for the phase constitution (see Figure 9a). The composition of the respective phase fractions is, however, quite distinct, as the Cu-rich FCC phase is more pronounced within the PBF-EB/M-manufactured samples without heat-treatment (see Table 6). The peak fraction of the Fe-Cr-enriched BCC phase in samples treated at 700 °C is in line with the results of the related XRD examination, revealing the highest peak intensity at an angle of 44.375°. Other examined phases show minor fractions with only marginal changes during heat-treatment and are therefore excluded from the presented results as well.

The examined main phases FCC and BCC (A2) exhibit a mixture of predominantly medium-sized grains (≤7 µm), a minority of nano-sized grains (≤1 µm), and an almost neglectable fraction of coarse grains (>7 µm) (see Figure 8d). Unlike the grain size evolution in the PBF-LB/M-manufactured samples, a clear indication of recrystallization cannot be drawn, as the grain size remains comparatively stable over all heat-treatment temperatures. Consequently, grain growth and Cu segregation already occur during processing (this being conducted at about 1000 °C), leading to a more thermally stabilized microstructure during subsequent heat-treatment.

The EBSD investigations demonstrate that the crystal orientation for all examined samples appears fragmented and no obvious preferred orientation can be detected (see Figure 9b). The angle of misorientation in the PBF-EB/M samples between the FCC and BCC (A2) phases shows a major peak at 43.03°, while the majority of the GOS is below 1°, indicating a low level of strain heterogeneities as well. Even though the IPF and PF results demonstrate more dense and localized patterns for samples heat-treated below 900 °C, the existence of an OR as formulated in [10,11,12,13,14] cannot be verified. While the FCC phase shows higher amounts of LAGBs, no obvious signs of twinning can be observed. Like the PBF-LB/M-manufactured samples, the PBF-EB/M counterparts show a majority of HAGBs, while the LAGB fraction falls between those measured in the samples of the P17 and re-scan conditions.

It can generally be stated that the cooling rate within the PBF-EB/M process is lower than that in PBF-LB/M [20], where in PBF-EB/M the entire build volume cools down slowly after processing of the uppermost layer. Phase maps revealed main phase fractions of FCC and BCC (A2), with a more distinct element distribution (see Table 7). Therefore, it can be presumed that the mismatch in thermal conductivity between the two phases is pronounced, leading to an enhanced difference in LAGB fractions within NT samples. This suggests that the reduced thermal gradients and the high build envelop temperature during PBF-EB/M promote partial homogenization during processing, thereby limiting additional recovery or recrystallization during subsequent heat-treatment. Other expectations, as discussed for PBF-LB/M-manufactured samples, cannot be fully confirmed.

Although the phase constitution within both manufacturing processes is similar, the phase fractions, grain size and grain morphology differ significantly. Additionally, the tested PBF-LB/M process conditions show specific differences within the resulting microstructure that need to be evaluated. These differences persist even after heat-treatment and may directly affect mechanical performance. To obtain an initial impression of the mechanical properties, the Vickers hardness was tested for all samples and heat-treatment conditions.

**Figure 10:**
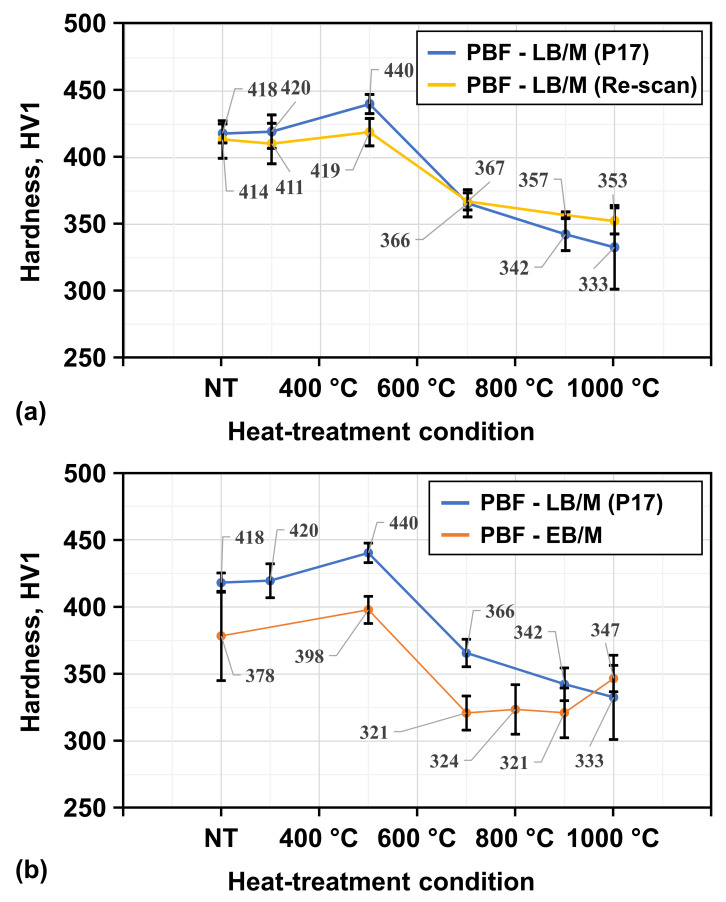
Vickers hardness (HV1) of (**a**) the PBF-LB/M P17 and re-scan conditions, as well as (**b**) the PBF-LB/M and PBF-EB/M samples.

The hardness of the PBF-LB/M- (see Figure 10a) and PBF-EB/M-manufactured (see Figure 10b) samples follows the same trend for heat-treatment temperatures up to 700 °C. Higher temperatures of 900 °C and 1000 °C led to partially increased hardness within PBF-EB/M- and a further decrease in PBF-LB/M-manufactured samples. Comparing the two conditions of the PBF-LB/M process shows that within experimental scatter the hardness of both process conditions is similar. Discrepancies in the hardness evolution within heat-treatment regimens above 700 °C can be addressed by slight differences in recrystallization and dislocation density. According to [4], the major factor influencing the strength of a material is the fraction of harder phases, i.e., BCC phases, within the sample, which is generally expected to result in higher hardness values. However, the results obtained in the present study indicate deviations from this simplified correlation. Therefore, a closer look at possible strengthening mechanisms will be taken, while eliminating non-fitting mechanisms to deduce the most likely explanation for the observed mechanical properties. The strength and, by extension, hardness of an alloy can be characterized by a superposition of different strengthening effects [9,22]. These effects can be attributed to various factors, including the elemental distribution in the form of the lattice distortion and solid solution, dislocation density, grain size and morphology, emerging precipitates, as well as the twinning or phase transformations during deformation. Results of the phase constitution evaluation and other examined factors pointed towards diverting characteristics. These need to be addressed for assessment here.

As demonstrated in Table 4, the BCC phase fraction of the PBF-LB/M-manufactured samples decreased by more than 10 wt. % between 700 °C and 900 °C, while it decreased only about 1.5 wt. % between 500 °C and 700 °C. Nevertheless, as displayed in Figure 10a, the hardness decline is about three times larger for the latter one. Similar behavior is observed for samples in the re-scan condition. Furthermore, the PBF-EB/M samples treated at 500 °C reveal a reduced fraction of the BCC phase but a higher hardness. Additionally, the grain size evolution seen would lead to the expectation of a more pronounced hardness reduction between 700 °C and 900 °C. These observations lead to the necessity of additional explanation that will be attempted at a later stage of the present paper.

While the hardness difference between the PBF-LB/M- and PBF-EB/M-processed samples in the lower temperature regimes can be associated with the respective grain size and FCC phase fraction, higher temperatures ≥ 900 °C have revealed BCC phase fractions within PBF-EB/M samples that contradict the observed hardness evolution. These apparent discrepancies indicate that the hardness evolution in the investigated HEA can only partially be explained by the phase fractions and grain size alone and that additional strengthening mechanisms must exist to explain the evaluated differences. According to [4,5,23], additions of small Cu fractions within an alloy can create distinct strengthening effects. It has been reported that Cu-rich nano-precipitation within interdendritic regions can promote a significant strengthening effect in similar compositions. These nano-precipitates cannot be resolved by the techniques used in the present study. However, the considerations detailed before and the evolution of hardness seen here point indirectly at the (only possible remaining) hardening effect through precipitation.

Higher heat-treatment temperatures promote the growth of Cu-rich precipitates along HAGBs [6]. This can explain the previously examined hardness trend, with the highest hardness observed for samples treated at 500 °C. This leads to the hypothesis that pronounced segregation of nano-sized Cu-rich precipitates at grain and sub-grain boundaries results in a strengthening effect that is later diminished by accumulated Cu segregations in samples treated above 500 °C.

The examined PBF-EB/M-manufactured samples already showed higher fractions of the Cu-rich FCC phase within the NT samples, thereby demonstrating a peak hardness at similar heat-treatment temperatures like PBF-LB/M, even though the EBSD phase maps reveal higher fractions of the BCC phase within samples treated at 700 °C. Other microstructural features such as the grain size and grain boundary characteristics would also lead to the assumption that the PBF-EB/M samples heat-treated at 700 °C should show a higher hardness than those heat-treated at 500 °C. However, the tested samples exhibit higher hardness and strength at 500 °C. This apparent contradiction indicates that conventional phase-fraction- or grain-size-based strengthening arguments alone cannot explain the observed behavior as well.

**Figure 11:**
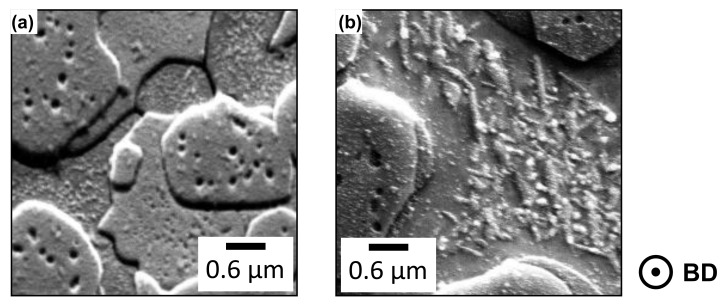
SE images of PBF-EB/M-manufactured samples of the following treatment conditions: (**a**) NT and (**b**) 500 °C with observed precipitates.

Therefore, EBSD investigations at a higher magnification have been applied, but did not reveal additional information sufficient to explain the hardness peak. Further observations of SE images simultaneously acquired during the EDS and EBSD investigations display structures within the Cu-rich FCC phase. These demonstrate similar shapes like precipitations described as Widmannstätten-type in [9]. These structures exist in PBF-EB/M-manufactured samples treated up to 700 °C and disappear at higher temperatures. While these Widmannstätten-like-shaped structures can be observed in higher density and nano-sized at 500 °C (see Figure 11b), heat-treatment at 700 °C leads to a decrease of the fraction accompanied by a partially increased size. NT samples also exhibit extensive precipitations within the Cu-rich FCC phase, but in contrast to former observations, a clear morphology cannot be confirmed (see Figure 11a).

The presented SE images reveal additional precipitations within the Fe-Cr-rich BCC (A2) phase. As these appear as voids on the prepared surface, it is likely that they detached during sample preparation and therefore cannot be further investigated in their current state. This indicates that alternative preparation routes, such as a focus ion beam (FIB) technique, are required for future investigations. Previously discussed observations indicate that both main phases exhibit nano-sized precipitations, similar to those reported in other multi-phase alloy systems [24]. Unfortunately, EBSD examinations and EDS analysis of all mentioned structures could not provide reliable, distinct compositional results. Therefore, further investigations by transmission electron microscopy (TEM), including selected area electron diffraction (SAED), would be necessary, but are beyond the scope of this study.

Direct comparison of the hardness of the tested samples with the existing literature shows that similar element combinations exist, although they are not completely identical. In particular, higher Cu fractions than those investigated within the present study are rarely reported, especially in combination with tensile testing data. Regardless, most studies point towards the importance of Cu, noting that small additions can create a strengthening effect via nano-precipitation and grain refinement, while higher fractions may reduce mechanical performance due to segregation [4]. Therefore, Cu is used as one criterion to distinguish the alloys, while the Al content is a second criterion, because Al has been reported to shift the phase composition toward higher BCC fractions [3].

A composition with the closest Cu fraction and a similar element combination has been reported in [2], where higher Mn fractions are used to hinder the segregation of a Cu-rich phase. Further additions of Al promoted a second emerging BCC phase accompanied by a dissolving FCC phase and increased hardness up to 436 ± 17 HV for Al_0.8_. This is consistent with the values of PBF-LB/M-manufactured samples heat-treated at 500 °C reported in the present work, while PBF-EB/M samples treated at the same temperature demonstrate hardness values similar to the Al_0.5_ alloy of [2]. Ultimately, this indicates that the manufacturing route and post-treatment can promote hardness levels similar to compositions with higher Al fractions. Other compositions reported in [3,4] were designed with a much lower Cu content to further suppress its segregation. These studies show that similar Al additions result in phase constitutions and hardness values as reported here for the PBF-LB/M samples heat-treated at 500 °C, finally demonstrating that the tested samples do not exhibit pronounced softening despite their higher Cu content.

This can be linked to results reported in [25]. HEAs of varying Cr fractions showed that Cr content promotes a Fe-Cr-rich BCC phase and leads to a significant hardness increase even without major changes in the phase constitution. Therefore, it can be presumed that the high Cr mass fraction (≥30%) in the tested composition provides a compensating strengthening contribution that offsets the potential softening effects of a higher Cu content.

**Figure 12:**
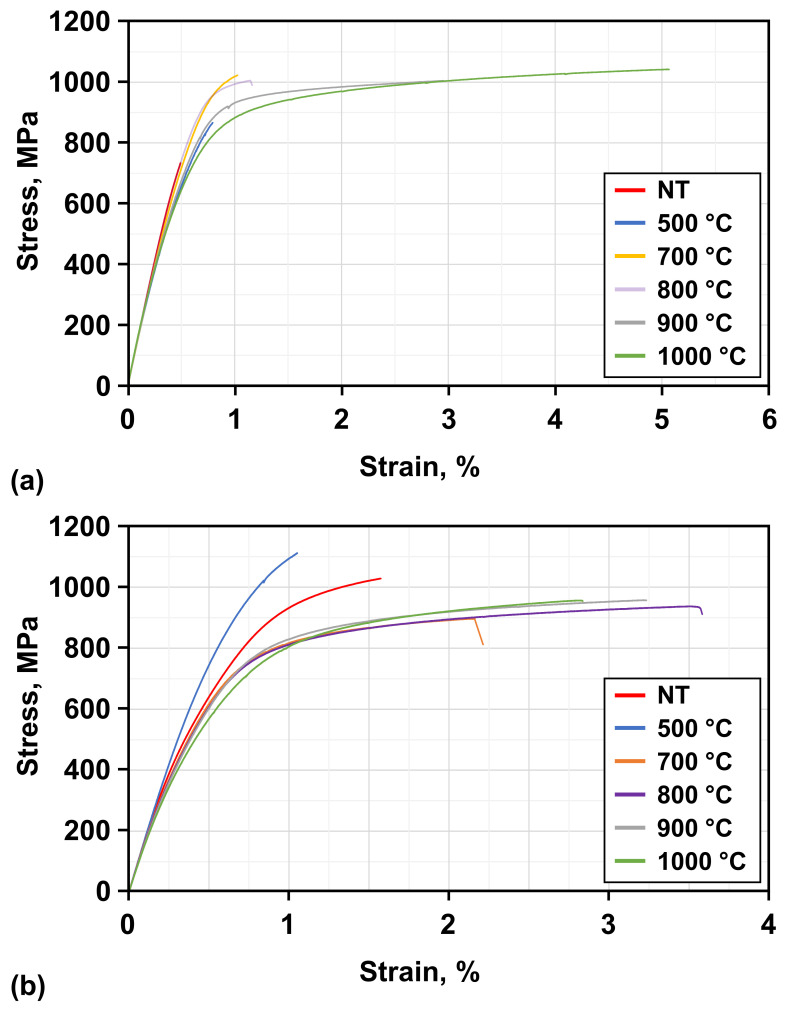
Stress–strain response of FeCrMnNiAlCu in different heat-treated conditions. (**a**) PBF-LB/M (P17), (**b**) PBF-EB/M.

Further on, the results of tensile testing are considered (see Figure 12a,b) and put into perspective with the measured hardness. A general observation is that the tested compositions manufactured by the applied AM processes suffer from significant brittleness. Therefore, certain samples could not be evaluated as they failed to deform plastically before failure (see Figure 12). While the evaluable results are listed in Table 8, the measured ultimate tensile strength (UTS) of most PBF-LB/M and some PBF-EB/M samples is highly unreliable due to brittleness and premature failure, respectively. An exception is made for examined samples revealing fracture elongation ≥ 2.0%. Nevertheless, all results reported in the case of UTS have to be evaluated with caution.

PBF-LB/M-manufactured samples can only be evaluated after heat-treatment at 700 °C and above, whereas the PBF-EB/M process reveals measurable deformation in all tested samples. This can be related to the measured hardness values, which are higher for PBF-LB/M-manufactured samples, as well as the respective SEM EBSD examinations. PBF-EB/M-manufactured samples show higher fractions of the FCC phase throughout all of the tested conditions, which can in part lead to lower hardness and higher ductility.

Nevertheless, as already hypothesized for the hardness evolution under heat-treatment, nano-sized precipitation and additional microstructural factors can influence the mechanical properties of the tested samples. Another possible influencing factor on the strength could be the chemical complexity and local compositional fluctuations within the Cu-rich FCC phase [26]. In reference to the aforementioned elemental distributions within the Cu-rich FCC phase, recent studies [24] have stated that chemical interfaces within phases can act as additional obstacles to dislocation motion.

Even though the UTS cannot be evaluated, it has to be taken into account that even though critical failure occurs during plastic deformation, parameters such as yield strength can still be evaluated. PBF-LB/M-manufactured samples demonstrate a decreasing yield strength for samples heat-treated above 800 °C, while those of the P17 variation treated at 700 °C and 800 °C exhibit a similar yield strength. The decreasing trend is thought to be rationalized by increasing grain size (due to recrystallization) as well as the increased fraction of the FCC phase. As mentioned before, no obvious signs of twinning-induced plasticity (TWIP) or transformation-induced plasticity (TRIP) effects have been observed for any of the tested samples. This has led to the assumption that the major influencing factor is the evolution of nano-sized precipitates (all other potential strengthening mechanisms could be eliminated through the results obtained in present work). Even though these structures could not be directly resolved and assessed by the applied methods (EDS, XRD and EBSD), SE images at least could reveal the existence of nano-scale microstructural features. A direct comparison of the examined hardness to the respective yield strength reveals similar results for samples treated at 500 °C, demonstrating the highest yield strength. In reference to the PBF-LB/M process, samples treated at 700 °C and 800 °C show similar yield strengths followed by a continued decrease in the measured values.

**Figure 13:**
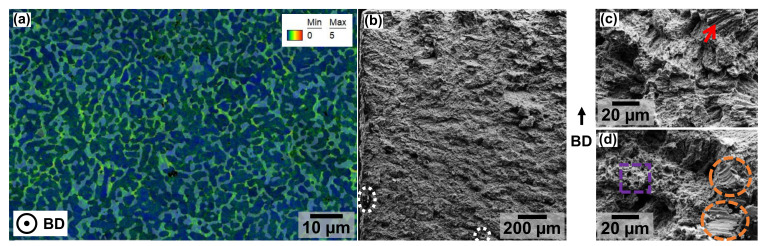
(**a**) KAM map of PBF-EB/M-manufactured samples with heat-treatment at 900 °C, (**b**) fractured surface with marked particles (white), (**c**) fractured surface with long shear bands (red), (**d**) fractured surface with dimples (purple) and cleavage surface (orange). The building direction is marked (KAM map shows cross-section of x-y plane, while fracture analysis was done in cross-section of x-z-plane).

The declining trend can be explained in a similar way to the hardness evolution, except for the sample heat-treated at 1000 °C. Nevertheless, as reported in other studies [27], the investigated multi-phase microstructure of the tested material is characterized by a heterogeneous deformation behavior (see Figure 13a). The gauge section of a sample heat-treated at 900 °C demonstrates a majority of dislocations residing within the Cu-rich FCC phase (bright background shows FCC phase fraction), as derived from the KAM map shown. Furthermore, the fracture surfaces indicate that phase-boundary-controlled dislocation accumulation contributes to additional strain hardening during plastic deformation [27].

Other microstructural characteristics such as the grain size and phase fractions, which influence the resulting yield strength, can be used to partially validate the measured values. SEM SE images of the fractured surfaces exhibit only minor quantities of defects such as unmolten particles or pores (see Figure 13b). Higher-magnification observations (see Figure 13c,d) clearly show fracture patterns similar to those reported in [3,4], including refined and distributed shear bands, long shear bands, cleavage surfaces associated with brittle behavior, as well as dimples associated with ductile deformation [3,4]. Finally, it has to be noted that even with an FCC phase fraction of nearly 40%, the fracture surfaces still show predominantly brittle material behavior.

## 4. Summary and Conclusions

The present work investigated the microstructural evolution and mechanical behavior of a Cu-containing multi-phase FeCrMnNiAl alloy manufactured by PBF-LB/M and PBF-EB/M, considering different scan strategies and post-process heat-treatments. Microstructural characterization was combined with hardness and tensile testing to establish structure–property relations. From the findings presented, the following conclusions can be drawn:

1. PBF-LB/M and PBF-EB/M processing resulted in specific phase fractions and grain morphologies, with PBF-EB/M promoting the evolution of a higher fraction of the Cu-rich FCC phase and a more equiaxed grain structure, while PBF-LB/M led to finer and more elongated grains.
2. Heat-treatment induced recovery and recrystallization in both manufacturing conditions established by PBF-LB/M, yet the evolution of low- and high-angle grain boundaries differed due to process-specific thermal histories and phase-dependent thermal conductivity.
3. Hardness evolution in PBF-LB/M samples could not be explained solely by BCC phase fraction or grain size. Instead, nano-sized Cu-rich precipitates formed at grain and sub-grain boundaries after low-temperature heat-treatment are thought to be the dominant strengthening mechanism.
4. Increasing heat-treatment temperature in PBF-LB/M-manufactured samples caused coarsening and increased segregation of Cu-rich precipitates, eventually leading to reduced hardness and strength.
5. A majority of the PBF-EB/M samples exhibited systematically lower hardness but higher ductility than the PBF-LB/M samples, consistent with their higher FCC phase fraction and all other relevant microstructural features.
6. Tensile testing revealed pronounced brittleness in most conditions, limiting the reliable determination of tensile properties like ultimate tensile strength; nevertheless, yield strength trends agreed with the hardness evolution and microstructural observations.

This work has shown the necessity of an overall approach for studying high-entropy alloys. General assumptions like the negative effects of a segregated Cu-phase cannot be confirmed. Variations in manufacturing processes, post-processing, and the resulting phase or elemental distribution can have a tremendous influence on the resulting material properties. Even though the achieved performance in tensile testing of the manufactured samples is still not fully reliable, the results elaborated already show potential steps to overcome shortcomings related to this kind of HEA composition.

## Figures and Tables

**Table 1:** Chemical composition (in wt. %) of the pre-alloyed powder used in present work.

Element	Fe	Cr	Mn	Ni	Al	Cu
**Content [wt. %]**	14.1	30.8	12.6	14.4	4.2	22.9

**Table 2:** Process parameters used for manufacturing of the FeCrMnNiAlCu alloy via PF-LB/M.

Variation	Laser Profile	Power, P [W]	Scanning Speed, v [mm/s]	Volumetric Energy Density, E_V_ [J/mm^3^]
P17	Gauss	300	725	73.9
Re-scan	Gauss	300	725	73.9
	Flat-Top	1000	2600	68.7

**Table 3:** Process parameters used for manufacturing of the FeCrMnNiAlCu alloy via PF-EB/M.

Acc. Voltage, V_a_ [kV]	Cathode Current, I_c_ [mA]	Scanning Speed, v [mm/s]	Volumetric Energy Density, E_V_ [J/mm^3^]
60	9	1375	70.1

**Table 4:** Evaluated phase fractions of PBF-LB/M-manufactured samples with and without re-scanning procedure.

Phase Composition (Fractions in %) for Different Heat-Treatment Conditions of PBF-LB/M P17 Samples						
Phases	NT	300 °C	500 °C	700 °C	900 °C	1000 °C
**Fe-Cr-rich BCC (A2) phase**	91.07	91.32	89.19	88.86	74.79	64.64
**Copper**	6.42	7.03	9.66	9.68	23.38	34.75
**Phase Composition (Fractions in %) for Different Heat-Treatment Conditions of PBF/LB/M Re-Scan Samples**						
**Phases**	**NT**	**300 °C**	**500 °C**	**700 °C**	**900 °C**	**1000 °C**
**Fe-Cr-rich BCC (A2) phase**	87.35	86.17	87.21	85.46	72.26	70.62
**Copper**	11.83	13.1	11.93	13.79	26.33	28.79

**Table 5:** Elemental distribution within the different phases of the PBF-LB/M-manufactured samples in different heat-treatment conditions (in wt. %).

Sample Definition (PBF-LB/M)		Elemental Distribution [wt. %]					
HT State	Phase Fraction	Fe	Cr	Ni	Al	Cu	Mn
**NT**	**BCC (A2)**	22.62	57.64	5.24	3.08	0.22	11.20
	**FCC**	7.56	19.39	3.26	5.34	47.23	17.22
**300 °C**	**BCC (A2)**	23.16	56.66	5.18	3.23	0.53	11.10
	**FCC**	5.67	17.31	4.10	2.85	57.33	12.54
**500 °C**	**BCC (A2)**	24.12	57.93	3.21	3.39	0.36	11.01
	**FCC**	6.27	19.90	2.78	2.66	55.02	13.38
**700 °C**	**BCC (A2)**	27.06	55.37	1.70	3.22	0.73	11.91
	**FCC**	3.52	10.88	2.39	2.75	67.20	13.26
**900 °C**	**BCC (A2)**	23.63	59.13	5.21	2.83	1.44	7.76
	**FCC**	1.86	5.38	12.64	5.83	62.69	11.60
**1000 °C**	**BCC (A2)**	23.79	67.49	0.10	1.36	0.20	7.06
	**FCC**	4.34	4.93	17.80	6.08	52.57	14.29

**Table 6:** Evaluated phase fractions of the PBF-EB/M-manufactured samples.

Phase Composition (Fractions In %) For Different Heat-Treatment Conditions of PBF-EB/M Samples						
Phases	NT	500 °C	700 °C	800 °C	900 °C	1000 °C
**Fe-Cr-rich BCC (A2) phase**	63.56	60.41	74.67	65.5	60.49	54.3
**Copper**	35.87	39.09	24.74	33.39	38.91	45.07

**Table 7:** Elemental distribution within the prevailing phases of PBF-EB/M-manufactured samples in different heat-treatment conditions (in wt. %).

Sample Definition (PBF-LB/M)	Elemental Distribution [wt. %]						
Heat-Treatment Condition	Phase Fraction	Fe	Cr	Ni	Al	Cu	Mn
**NT**	**BCC (A2)**	24.38	68.76	0.46	2.30	0.00	4.10
	**FCC**	1.33	1.08	10.74	4.12	76.85	5.87
**500 °C**	**BCC (A2)**	24.82	68.42	1.22	1.41	0.00	4.12
	**FCC**	1.03	0.94	10.05	4.83	76.46	6.69
**700 °C**	**BCC (A2)**	24.02	69.16	0.13	2.13	0.00	4.55
	**FCC**	0.44	1.04	11.15	5.76	74.71	6.91
**800 °C**	**BCC (A2)**	18.74	77.75	0.40	1.53	0.00	1.57
	**FCC**	0.31	0.69	10.54	4.70	76.84	6.93
**900 °C**	**BCC (A2)**	25.62	67.79	0.29	1.44	0.00	4.85
	**FCC**	0.92	2.27	14.16	5.51	67.71	9.43
**1000 °C**	**BCC (A2)**	22.91	72.21	0.17	1.71	0.42	2.58
	**FCC**	3.64	3.88	16.12	7.57	61.85	6.94

**Table 8:** Results of heat-treated samples obtained by tensile testing.

PBF-LB/M	P17				Re-Scan	
Heat-Treatment Condition	700 °C	800 °C	900 °C	1000 °C	900 °C	1000 °C
**Yield Strength** **[MPa]**	935 ± 18.0	949.3 ± 10.2	854.2 ± 28.9	763.3 ± 4.7	863.3 ± 24.9	779.3 ± 29.2
**Ultimate Tensile Strength** **[MPa]**	977.6 ± 30.9	999.8 ± 20.5	1001.6 ± 17.0	1039.8 ± 6.2	984.8 ± 15.3	949.7 ± 127.5
**Elongation at** **Fracture** **[%]**	0.3 ± 0.1	0.6 ± 0.3	2.1 ± 0.3	4.0 ± 0.2	1.2 ± 0.3	2.5 ± 1.6
**PBF-EB/M**						
**Heat-Treatment** **Condition**	**NT**	**500 °C**	**700 °C**	**800 °C**	**900 °C**	**1000 °C**
**Yield Strength** **[MPa]**	783.3 ± 30.9	970.0 ± 17.3	718.8 ± 26.5	741.7 ± 28.7	698.3 ± 8.5	683.3 ± 36.8
**Ultimate Tensile Strength** **[MPa]**	1029.4 ± 13.2	1104.1 ± 18.3	913.6 ± 11.9	903.4 ± 33.1	954.6 ± 2.4	936.6 ± 42.5
**Elongation at** **Fracture** **[%]**	1.0 ± 0.2	0.4 ± 0.1	1.6 ± 0.4	2.4 ± 1.4	2.9 ± 0.4	2.3 ± 0.5

## Data Availability

The original contributions presented in this study are included in the article. Further inquiries can be directed to the corresponding authors.
